# 8-Quinolyl 5-(dimethyl­amino)­naphthalene-1-sulfonate

**DOI:** 10.1107/S160053681002979X

**Published:** 2010-07-31

**Authors:** Zuo-an Xiao, Dan Zhan

**Affiliations:** a School of Chemical Engineering and Food Science, Xiangfan University, Xiangfan 441053, People’s Republic of China

## Abstract

In the title compound, C_21_H_18_N_2_O_3_S, the dihedral angle between the naphthalene and quinoline ring systems is 55.53 (2)°, and the torsion angle involving the connecting C—S—O—C atoms is 87.60 (3)°. In the crystal structure, weak inter­molecular C—H⋯O hydrogen bonds connect mol­ecules into chains along [100] and there are π–π stacking inter­actions between pairs of chains with a centroid–centroid distance of 3.5485 (15) Å.

## Related literature

For background information and the applications of compounds containing the 5-(dimethyl­amino)­naphthalene-1-sulfonyl group, see: Li *et al.* (1975 ▶); Walkup & Imperiali (1997 ▶); Chen & Chen (2004 ▶).
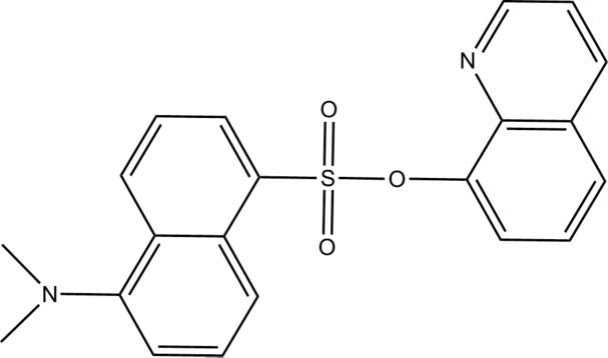


## Experimental

### 

#### Crystal data

C_21_H_18_N_2_O_3_S
*M* *_r_* = 378.43
Triclinic, 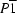

*a* = 9.5556 (12) Å
*b* = 10.1237 (12) Å
*c* = 11.4182 (14) Å
α = 108.736 (2)°
β = 100.426 (2)°
γ = 111.860 (2)°
*V* = 912.30 (19) Å^3^
*Z* = 2
Mo *K*α radiation
μ = 0.20 mm^−1^
*T* = 298 K
0.20 × 0.20 × 0.20 mm

#### Data collection

Bruker SMART CCD diffractometer
Absorption correction: multi-scan (*SADABS*; Sheldrick, 1997 ▶) *T*_min_ = 0.961, *T*_max_ = 0.980
5269 measured reflections
3526 independent reflections
2959 reflections with *I* > 2σ(*I*)
*R*_int_ = 0.054

#### Refinement

*R*[*F* ^2^ > 2σ(*F* ^2^)] = 0.050
*wR*(*F* ^2^) = 0.132
*S* = 1.04
3526 reflections
246 parameters
H-atom parameters constrained
Δρ_max_ = 0.28 e Å^−3^
Δρ_min_ = −0.34 e Å^−3^

### 

Data collection: *SMART* (Bruker, 2007 ▶); cell refinement: *SAINT* (Bruker, 2007 ▶); data reduction: *SAINT*; program(s) used to solve structure: *SHELXS97* (Sheldrick, 2008 ▶); program(s) used to refine structure: *SHELXL97* (Sheldrick, 2008 ▶); molecular graphics: *PLATON* (Spek, 2009 ▶); software used to prepare material for publication: *SHELXTL* (Sheldrick, 2008 ▶).

## Supplementary Material

Crystal structure: contains datablocks global, I. DOI: 10.1107/S160053681002979X/lh5093sup1.cif

Structure factors: contains datablocks I. DOI: 10.1107/S160053681002979X/lh5093Isup2.hkl

Additional supplementary materials:  crystallographic information; 3D view; checkCIF report

## Figures and Tables

**Table 1:** Hydrogen-bond geometry (Å, °)

*D*—H⋯*A*	*D*—H	H⋯*A*	*D*⋯*A*	*D*—H⋯*A*
C16—H16⋯O1^i^	0.93	2.52	3.411 (3)	160

Symmetry code: (i) 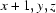.

**Fig. 1.**
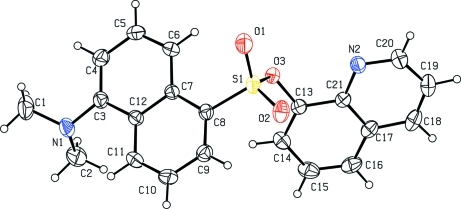
The molecular structure of (I), with displacement ellipsoids drawn at the 50% probability level.

**Fig. 2.**
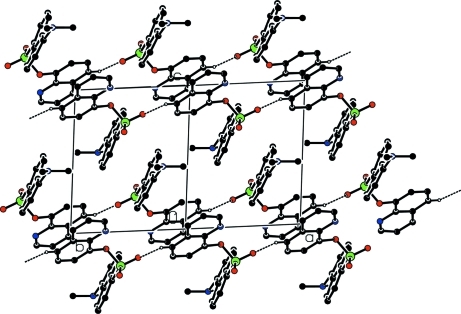
Part of the crystal structure of (I) showing hydrogen bonds as dashed lines.

## supplementary crystallographic information

### Comment

The dansyl fluorophore (5-(dimethylamino)naphthalene-1-sulfonyl) is characterized by a charge transfer excited state exhibiting solvatochromism and high emission quantum yields (Li *et al.*, 1975). These characteristics, together with the synthetic flexibility of the sulfonic acid group, have led the dansyl fluorophore to be a core-structure present in many fluorescent sensors and labels for the detection of both metal cations and anions (Walkup & Imperiali, 1997; Chen & Chen, 2004). We are interested in designing fluorescent drug or ligand analogs that are expected to bind to hydrophobic sites in proteins or membranes. With this mind, the title compound, (I), was prepared and we report the crystal stucture herein.

In the molecular structure (Fig. 1), the dihedral angle between the naphthalene and quinoline ring systems is 55.53 (2)°, and these aromatic ring ststems are connected by the atoms C8—S1—O3—C13, giving a torsion angle of 87.60 (3)°. In the crystal structure (Fig. 2) molecules are linked by weak intermolecular C—H···O hydrogen bonds forming 1-D chains along [100]. Pairs of chains are connected by weak π–π stacking interactions with Cg···Cg(2-x, -y, -z) = 3.5485 (15), where Cg is the centroid defined by ring atoms C13-C17/C21.

### Experimental

8-Hydroxyquinolin (0.16 g, 1 mmol) was added to a stirred solution of dansyl chloride (0.27 g, 1 mmol) in dry acetone (40 ml). The reaction mixture was allowed to stir for 12 hr at 293 K. The solvent was evaporated and the residue was purified by column chromatography (petroleun ether-ethyl acetate,1:4 *v*/*v*) to afford the title compound as a yellow solid. Single crystals suitable for X-ray diffraction were prepared by slow evaporation of a solution of the title compound in ethyl acetate at room temperature.

### Refinement

All H atoms were placed in idealized positions [*C*—*H*(methyl)=0.96 Å, and 0.93 Å (aromatic),with *U*_iso_(H)= 1.5*U*_eq_(methyl C) 1.2*U*_eq_(other C).

### Crystal data

**Table:**

C_21_H_18_N_2_O_3_S	*Z* = 2
*M**_r_* = 378.43	*F*(000) = 396
Triclinic, *P*1	*D*_x_ = 1.378 Mg m^−^^3^
Hall symbol: -P 1	Mo *K*α radiation, λ = 0.71073 Å
*a* = 9.5556 (12) Å	Cell parameters from 2502 reflections
*b* = 10.1237 (12) Å	θ = 1.7–22.5°
*c* = 11.4182 (14) Å	µ = 0.20 mm^−^^1^
α = 108.736 (2)°	*T* = 298 K
β = 100.426 (2)°	Block, yellow
γ = 111.860 (2)°	0.20 × 0.20 × 0.20 mm
*V* = 912.30 (19) Å^3^	

### Data collection

**Table:**

Bruker SMART CCD diffractometer	3526 independent reflections
Radiation source: fine-focus sealed tube	2959 reflections with *I* > 2σ(*I*)
graphite	*R*_int_ = 0.054
φ and ω scans	θ_max_ = 26.0°, θ_min_ = 2.0°
Absorption correction: multi-scan (*SADABS*; Sheldrick, 1997)	*h* = −11→11
*T*_min_ = 0.961, *T*_max_ = 0.980	*k* = −12→12
5269 measured reflections	*l* = −14→14

### Refinement

**Table:**

Refinement on *F*^2^	Primary atom site location: structure-invariant direct methods
Least-squares matrix: full	Secondary atom site location: difference Fourier map
*R*[*F*^2^ > 2σ(*F*^2^)] = 0.050	Hydrogen site location: inferred from neighbouring sites
*wR*(*F*^2^) = 0.132	H-atom parameters constrained
*S* = 1.04	*w* = 1/[σ^2^(*F*_o_^2^) + (0.0622*P*)^2^ + 0.1786*P*] where *P* = (*F*_o_^2^ + 2*F*_c_^2^)/3
3526 reflections	(Δ/σ)_max_ = 0.001
246 parameters	Δρ_max_ = 0.28 e Å^−^^3^
0 restraints	Δρ_min_ = −0.34 e Å^−^^3^

### Special details

**Table:**

Geometry. All e.s.d.'s (except the e.s.d. in the dihedral angle between two l.s. planes) are estimated using the full covariance matrix. The cell e.s.d.'s are taken into account individually in the estimation of e.s.d.'s in distances, angles and torsion angles; correlations between e.s.d.'s in cell parameters are only used when they are defined by crystal symmetry. An approximate (isotropic) treatment of cell e.s.d.'s is used for estimating e.s.d.'s involving l.s. planes.
Refinement. Refinement of *F*^2^ against ALL reflections. The weighted *R*-factor *wR* and goodness of fit *S* are based on *F*^2^, conventional *R*-factors *R* are based on *F*, with *F* set to zero for negative *F*^2^. The threshold expression of *F*^2^ > σ(*F*^2^) is used only for calculating *R*-factors(gt) *etc*. and is not relevant to the choice of reflections for refinement. *R*-factors based on *F*^2^ are statistically about twice as large as those based on *F*, and *R*- factors based on ALL data will be even larger.

### Fractional atomic coordinates and isotropic or equivalent isotropic displacement parameters (Å^2^)

**Table:**

	*x*	*y*	*z*	*U*_iso_*/*U*_eq_	
C1	0.3811 (4)	−0.3563 (4)	0.4404 (3)	0.0808 (9)	
H1A	0.3092	−0.3111	0.4357	0.121*	
H1B	0.3945	−0.3739	0.5185	0.121*	
H1C	0.3375	−0.4546	0.3645	0.121*	
C2	0.6414 (4)	−0.3214 (4)	0.4384 (3)	0.0776 (8)	
H2A	0.5946	−0.4150	0.3577	0.116*	
H2B	0.6564	−0.3480	0.5116	0.116*	
H2C	0.7433	−0.2490	0.4419	0.116*	
C3	0.5317 (3)	−0.1772 (2)	0.3569 (2)	0.0460 (5)	
C4	0.4046 (3)	−0.2372 (3)	0.2452 (2)	0.0549 (6)	
H4	0.3129	−0.3287	0.2251	0.066*	
C5	0.4103 (3)	−0.1632 (3)	0.1607 (2)	0.0543 (6)	
H5	0.3217	−0.2059	0.0857	0.065*	
C6	0.5421 (3)	−0.0303 (3)	0.1855 (2)	0.0469 (5)	
H6	0.5452	0.0133	0.1251	0.056*	
C7	0.6746 (2)	0.0422 (2)	0.30273 (19)	0.0386 (4)	
C8	0.8158 (2)	0.1871 (2)	0.34276 (19)	0.0407 (5)	
C9	0.9371 (3)	0.2558 (3)	0.4599 (2)	0.0498 (5)	
H9	1.0277	0.3491	0.4818	0.060*	
C10	0.9242 (3)	0.1849 (3)	0.5467 (2)	0.0593 (6)	
H10	1.0052	0.2328	0.6279	0.071*	
C11	0.7943 (3)	0.0466 (3)	0.5135 (2)	0.0550 (6)	
H11	0.7878	0.0016	0.5729	0.066*	
C12	0.6684 (2)	−0.0309 (2)	0.39138 (19)	0.0423 (5)	
C13	1.0085 (2)	0.1803 (2)	0.12403 (19)	0.0406 (4)	
C14	1.0695 (3)	0.1102 (3)	0.1871 (2)	0.0514 (5)	
H14	1.0128	0.0581	0.2302	0.062*	
C15	1.2188 (3)	0.1174 (3)	0.1866 (2)	0.0616 (6)	
H15	1.2622	0.0718	0.2315	0.074*	
C16	1.3009 (3)	0.1902 (3)	0.1213 (2)	0.0599 (6)	
H16	1.3999	0.1942	0.1221	0.072*	
C17	1.2370 (3)	0.2596 (3)	0.0523 (2)	0.0487 (5)	
C18	1.3112 (3)	0.3301 (3)	−0.0238 (2)	0.0623 (7)	
H18	1.4073	0.3321	−0.0306	0.075*	
C19	1.2415 (3)	0.3946 (3)	−0.0863 (3)	0.0643 (7)	
H19	1.2891	0.4411	−0.1366	0.077*	
C20	1.0978 (3)	0.3904 (3)	−0.0744 (2)	0.0584 (6)	
H20	1.0533	0.4377	−0.1164	0.070*	
C21	1.0888 (2)	0.2580 (2)	0.05481 (18)	0.0399 (4)	
N1	0.5359 (2)	−0.2493 (2)	0.4443 (2)	0.0572 (5)	
N2	1.0200 (2)	0.3242 (2)	−0.00774 (17)	0.0479 (4)	
O1	0.6994 (2)	0.2943 (2)	0.18886 (19)	0.0656 (5)	
O2	0.9897 (2)	0.42788 (18)	0.30555 (16)	0.0607 (4)	
O3	0.85533 (16)	0.16883 (17)	0.11789 (13)	0.0442 (4)	
S1	0.84187 (7)	0.28769 (6)	0.24117 (5)	0.04560 (19)	

### Atomic displacement parameters (Å^2^)

**Table:**

	*U*^11^	*U*^22^	*U*^33^	*U*^12^	*U*^13^	*U*^23^
C1	0.084 (2)	0.085 (2)	0.118 (2)	0.0459 (17)	0.0604 (19)	0.073 (2)
C2	0.081 (2)	0.089 (2)	0.100 (2)	0.0529 (17)	0.0357 (17)	0.0648 (19)
C3	0.0476 (12)	0.0491 (12)	0.0530 (12)	0.0252 (10)	0.0223 (10)	0.0291 (10)
C4	0.0446 (13)	0.0498 (13)	0.0624 (14)	0.0144 (10)	0.0138 (11)	0.0260 (11)
C5	0.0417 (12)	0.0600 (14)	0.0521 (13)	0.0197 (11)	0.0044 (10)	0.0239 (11)
C6	0.0462 (12)	0.0550 (13)	0.0448 (11)	0.0258 (10)	0.0115 (10)	0.0268 (10)
C7	0.0400 (11)	0.0433 (11)	0.0403 (10)	0.0227 (9)	0.0167 (9)	0.0212 (9)
C8	0.0438 (11)	0.0435 (11)	0.0417 (11)	0.0228 (9)	0.0208 (9)	0.0198 (9)
C9	0.0454 (12)	0.0483 (12)	0.0469 (12)	0.0162 (10)	0.0157 (10)	0.0165 (10)
C10	0.0515 (14)	0.0681 (15)	0.0400 (12)	0.0201 (12)	0.0035 (10)	0.0171 (11)
C11	0.0587 (14)	0.0654 (15)	0.0412 (12)	0.0257 (12)	0.0126 (10)	0.0289 (11)
C12	0.0443 (12)	0.0480 (11)	0.0427 (11)	0.0245 (10)	0.0167 (9)	0.0240 (9)
C13	0.0396 (11)	0.0424 (11)	0.0377 (10)	0.0196 (9)	0.0132 (9)	0.0138 (9)
C14	0.0632 (15)	0.0557 (13)	0.0456 (12)	0.0330 (12)	0.0221 (11)	0.0249 (10)
C15	0.0694 (17)	0.0730 (16)	0.0583 (14)	0.0493 (14)	0.0174 (13)	0.0295 (13)
C16	0.0451 (13)	0.0693 (15)	0.0659 (15)	0.0349 (12)	0.0163 (12)	0.0208 (13)
C17	0.0404 (11)	0.0451 (11)	0.0524 (12)	0.0182 (9)	0.0171 (10)	0.0124 (10)
C18	0.0472 (14)	0.0584 (14)	0.0719 (16)	0.0169 (11)	0.0325 (12)	0.0189 (13)
C19	0.0682 (17)	0.0630 (15)	0.0685 (16)	0.0237 (13)	0.0401 (14)	0.0347 (13)
C20	0.0682 (16)	0.0602 (14)	0.0589 (14)	0.0313 (13)	0.0294 (12)	0.0334 (12)
C21	0.0376 (11)	0.0390 (10)	0.0380 (10)	0.0170 (9)	0.0127 (8)	0.0114 (8)
N1	0.0615 (13)	0.0614 (12)	0.0719 (13)	0.0315 (10)	0.0320 (10)	0.0466 (11)
N2	0.0497 (11)	0.0542 (11)	0.0505 (10)	0.0262 (9)	0.0226 (9)	0.0291 (9)
O1	0.0683 (11)	0.0834 (12)	0.0966 (13)	0.0545 (10)	0.0485 (10)	0.0642 (11)
O2	0.0660 (11)	0.0423 (8)	0.0713 (10)	0.0186 (8)	0.0341 (9)	0.0231 (8)
O3	0.0389 (8)	0.0519 (8)	0.0446 (8)	0.0201 (7)	0.0182 (6)	0.0227 (7)
S1	0.0499 (3)	0.0456 (3)	0.0582 (3)	0.0270 (3)	0.0298 (3)	0.0300 (3)

### Geometric parameters (Å, °)

**Table:**

C1—N1	1.454 (3)	C10—H10	0.9300
C1—H1A	0.9600	C11—C12	1.412 (3)
C1—H1B	0.9600	C11—H11	0.9300
C1—H1C	0.9600	C13—C14	1.358 (3)
C2—N1	1.448 (3)	C13—O3	1.411 (2)
C2—H2A	0.9600	C13—C21	1.415 (3)
C2—H2B	0.9600	C14—C15	1.403 (3)
C2—H2C	0.9600	C14—H14	0.9300
C3—C4	1.364 (3)	C15—C16	1.360 (4)
C3—N1	1.417 (3)	C15—H15	0.9300
C3—C12	1.433 (3)	C16—C17	1.410 (3)
C4—C5	1.396 (3)	C16—H16	0.9300
C4—H4	0.9300	C17—C21	1.416 (3)
C5—C6	1.356 (3)	C17—C18	1.418 (3)
C5—H5	0.9300	C18—C19	1.353 (4)
C6—C7	1.413 (3)	C18—H18	0.9300
C6—H6	0.9300	C19—C20	1.391 (4)
C7—C12	1.430 (3)	C19—H19	0.9300
C7—C8	1.434 (3)	C20—N2	1.320 (3)
C8—C9	1.362 (3)	C20—H20	0.9300
C8—S1	1.766 (2)	C21—N2	1.363 (3)
C9—C10	1.396 (3)	O1—S1	1.4188 (17)
C9—H9	0.9300	O2—S1	1.4183 (17)
C10—C11	1.356 (3)	O3—S1	1.5933 (15)
			
N1—C1—H1A	109.5	C11—C12—C3	121.60 (18)
N1—C1—H1B	109.5	C7—C12—C3	119.32 (18)
H1A—C1—H1B	109.5	C14—C13—O3	120.52 (19)
N1—C1—H1C	109.5	C14—C13—C21	122.3 (2)
H1A—C1—H1C	109.5	O3—C13—C21	117.06 (17)
H1B—C1—H1C	109.5	C13—C14—C15	119.3 (2)
N1—C2—H2A	109.5	C13—C14—H14	120.3
N1—C2—H2B	109.5	C15—C14—H14	120.3
H2A—C2—H2B	109.5	C16—C15—C14	120.8 (2)
N1—C2—H2C	109.5	C16—C15—H15	119.6
H2A—C2—H2C	109.5	C14—C15—H15	119.6
H2B—C2—H2C	109.5	C15—C16—C17	120.5 (2)
C4—C3—N1	123.8 (2)	C15—C16—H16	119.7
C4—C3—C12	119.17 (18)	C17—C16—H16	119.7
N1—C3—C12	117.06 (19)	C16—C17—C21	119.7 (2)
C3—C4—C5	120.9 (2)	C16—C17—C18	123.8 (2)
C3—C4—H4	119.5	C21—C17—C18	116.5 (2)
C5—C4—H4	119.5	C19—C18—C17	119.8 (2)
C6—C5—C4	121.6 (2)	C19—C18—H18	120.1
C6—C5—H5	119.2	C17—C18—H18	120.1
C4—C5—H5	119.2	C18—C19—C20	119.0 (2)
C5—C6—C7	120.30 (19)	C18—C19—H19	120.5
C5—C6—H6	119.8	C20—C19—H19	120.5
C7—C6—H6	119.8	N2—C20—C19	124.7 (2)
C6—C7—C12	118.50 (18)	N2—C20—H20	117.6
C6—C7—C8	125.16 (18)	C19—C20—H20	117.6
C12—C7—C8	116.32 (18)	N2—C21—C13	119.30 (18)
C9—C8—C7	122.78 (18)	N2—C21—C17	123.32 (19)
C9—C8—S1	116.00 (16)	C13—C21—C17	117.37 (19)
C7—C8—S1	121.22 (15)	C3—N1—C2	113.78 (19)
C8—C9—C10	119.4 (2)	C3—N1—C1	116.1 (2)
C8—C9—H9	120.3	C2—N1—C1	110.5 (2)
C10—C9—H9	120.3	C20—N2—C21	116.59 (19)
C11—C10—C9	120.4 (2)	C13—O3—S1	117.87 (12)
C11—C10—H10	119.8	O2—S1—O1	119.57 (11)
C9—C10—H10	119.8	O2—S1—O3	108.77 (8)
C10—C11—C12	121.9 (2)	O1—S1—O3	104.37 (10)
C10—C11—H11	119.0	O2—S1—C8	109.32 (10)
C12—C11—H11	119.0	O1—S1—C8	110.89 (10)
C11—C12—C7	119.01 (19)	O3—S1—C8	102.41 (8)
			
N1—C3—C4—C5	−177.7 (2)	C16—C17—C18—C19	−179.7 (2)
C12—C3—C4—C5	3.9 (3)	C21—C17—C18—C19	1.6 (3)
C3—C4—C5—C6	0.6 (4)	C17—C18—C19—C20	0.1 (4)
C4—C5—C6—C7	−3.7 (4)	C18—C19—C20—N2	−1.5 (4)
C5—C6—C7—C12	2.2 (3)	C14—C13—C21—N2	179.21 (19)
C5—C6—C7—C8	−176.3 (2)	O3—C13—C21—N2	3.3 (3)
C6—C7—C8—C9	176.8 (2)	C14—C13—C21—C17	0.5 (3)
C12—C7—C8—C9	−1.7 (3)	O3—C13—C21—C17	−175.38 (17)
C6—C7—C8—S1	−3.1 (3)	C16—C17—C21—N2	179.12 (19)
C12—C7—C8—S1	178.40 (14)	C18—C17—C21—N2	−2.1 (3)
C7—C8—C9—C10	−1.0 (3)	C16—C17—C21—C13	−2.2 (3)
S1—C8—C9—C10	178.88 (17)	C18—C17—C21—C13	176.51 (18)
C8—C9—C10—C11	1.8 (4)	C4—C3—N1—C2	107.4 (3)
C9—C10—C11—C12	0.2 (4)	C12—C3—N1—C2	−74.3 (3)
C10—C11—C12—C7	−3.0 (3)	C4—C3—N1—C1	−22.5 (3)
C10—C11—C12—C3	179.9 (2)	C12—C3—N1—C1	155.8 (2)
C6—C7—C12—C11	−175.00 (19)	C19—C20—N2—C21	1.0 (4)
C8—C7—C12—C11	3.6 (3)	C13—C21—N2—C20	−177.77 (19)
C6—C7—C12—C3	2.2 (3)	C17—C21—N2—C20	0.9 (3)
C8—C7—C12—C3	−179.18 (17)	C14—C13—O3—S1	82.5 (2)
C4—C3—C12—C11	171.9 (2)	C21—C13—O3—S1	−101.55 (17)
N1—C3—C12—C11	−6.6 (3)	C13—O3—S1—O2	28.05 (16)
C4—C3—C12—C7	−5.2 (3)	C13—O3—S1—O1	156.75 (14)
N1—C3—C12—C7	176.32 (17)	C13—O3—S1—C8	−87.57 (15)
O3—C13—C14—C15	177.12 (19)	C9—C8—S1—O2	−0.92 (19)
C21—C13—C14—C15	1.4 (3)	C7—C8—S1—O2	178.98 (15)
C13—C14—C15—C16	−1.6 (4)	C9—C8—S1—O1	−134.83 (17)
C14—C15—C16—C17	−0.2 (4)	C7—C8—S1—O1	45.06 (19)
C15—C16—C17—C21	2.1 (3)	C9—C8—S1—O3	114.31 (17)
C15—C16—C17—C18	−176.5 (2)	C7—C8—S1—O3	−65.80 (17)

### Hydrogen-bond geometry (Å, °)

**Table:**

*D*—H···*A*	*D*—H	H···*A*	*D*···*A*	*D*—H···*A*
C16—H16···O1^i^	0.93	2.52	3.411 (3)	160

Symmetry codes: (i) *x*+1, *y*, *z*.
